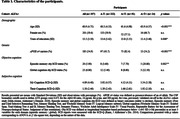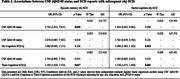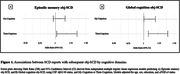# The relationship between dyadic perspective in subjective reports and objective cognitive decline in preclinical Alzheimer's

**DOI:** 10.1002/alz.087054

**Published:** 2025-01-03

**Authors:** David López‐Martos, Anna Brugulat‐Serrat, Alba Cañas‐Martínez, Lidia Canals‐Gispert, Paula Marne, Nina Gramunt, Marc Suárez‐Calvet, Marta Milà‐Alomà, Carolina Minguillon, Karine Fauria, Henrik Zetterberg, Kaj Blennow, Jose Luis Molinuevo, Juan Domingo Gispert, Oriol Grau‐Rivera, Gonzalo Sánchez‐Benavides, for the ALFA study

**Affiliations:** ^1^ Barcelonaβeta Brain Research Center (BBRC), Pasqual Maragall Foundation, Barcelona Spain; ^2^ Hospital del Mar Research Institute (IMIM), Barcelona Spain; ^3^ Global Brain Health Institute, San Francisco, CA USA; ^4^ Hospital del Mar Medical Research Institute (IMIM), Barcelona Spain; ^5^ Centro de Investigación Biomédica en Red de Fragilidad y Envejecimiento Saludable (CIBERFES), Instituto de Salud Carlos III, Madrid Spain; ^6^ Pasqual Maragall Foundation, Barcelona Spain; ^7^ Servei de Neurologia, Hospital del Mar, Barcelona Spain; ^8^ Department of Radiology and Biomedical Imaging, University of California, San Francisco, San Francisco, CA USA; ^9^ Department of Veterans Affairs Medical Center, Northern California Institute for Research and Education (NCIRE), San Francisco, CA USA; ^10^ Barcelonaβeta Brain Research Center (BBRC), Barcelona Spain; ^11^ Centro de Investigación Biomédica en Red de Fragilidad y Envejecimiento Saludable (CIBERFES), Madrid Spain; ^12^ Department of Neurodegenerative Disease, UCL Queen Square Institute of Neurology, University College London, London, ‐ United Kingdom; ^13^ Wisconsin Alzheimer’s Disease Research Center, School of Medicine and Public Health, University of Wisconsin‐Madison, Madison, WI USA; ^14^ Hong Kong Center for Neurodegenerative Diseases, Clear Water Bay Hong Kong; ^15^ Institute of Neuroscience and Physiology, University of Gothenburg, Mölndal Sweden; ^16^ UK Dementia Research Institute at UCL, London United Kingdom; ^17^ Department of Psychiatry and Neurochemistry, Institute of Neuroscience and Physiology, The Sahlgrenska Academy at the University of Gothenburg, Mölndal Sweden; ^18^ Clinical Neurochemistry Laboratory Sahlgrenska University Hospital, Mölndal Sweden; ^19^ Department of Psychiatry and Neurochemistry, Institute of Neuroscience and Physiology, University of Gothenburg, Mölndal Sweden; ^20^ Centro de Investigación Biomédica en Red Bioingeniería, Biomateriales y Nanomedicina (CIBER‐BBN), Instituto de Salud Carlos III, Madrid Spain; ^21^ Hospital del Mar Research Institute, Barcelona, Barcelona Spain

## Abstract

**Background:**

Subjective Cognitive Decline (SCD) may represent the initial symptom of Alzheimer’s disease (AD), but SCD may be absent and/or unrelated to actual cognitive decline. Objective Subtle Cognitive Decline (obj‐SCD) can be identified through longitudinal standardized neuropsychological tests in individuals not yet meeting criteria for Mild Cognitive Impairment (MCI). We argue that the relationship between SCD and obj‐SCD might help to inform clinical and research criteria in pre‐MCI stages. This study explores the dyadic perception (participant‐informant) of SCD as an early symptomatic marker of obj‐SCD in Cognitively Unimpaired (CU) population at increased risk of (AD) dementia.

**Method:**

Three hundred thirty‐seven CU participants from the ALFA+ prospective cohort study (with three‐year follow‐up) were included. AT(N) profiles were defined at baseline with Cerebrospinal Fluid (CSF) biomarkers. Baseline reports of SCD, “My‐Cognition” for the participant and “Their‐Cognition” for the informant, were measured with the SCD‐Questionnaire (SCD‐Q). AD biomarker‐based reliable change indices adjusted for practice effect (A‐T‐[N]‐ group’s longitudinal performance as reference) were computed for the robust measurement of cognitive trajectory. Considering the relationship between the number of neuropsychological measures and the base rate of impaired scores, obj‐SCD was defined as longitudinal biomarker‐based performance below ‐1.645 SD (<5^th^‐percentile) in at least 3 variables within each domain (episodic memory | global). Episodic memory and global obj‐SCD were analyzed using logistic linear regression with CSF Aβ42/40 status and SCD reports as predictors.

**Result:**

CSF Aβ‐status was not associated with global, but with memory obj‐SCD (OR = 2.743, 95%CI = 1.013‐7.811). SCD scores in My‐Cognition and Their‐Cognition were associated with global obj‐SCD (OR = 1.096, 95%CI = 1.003‐1.192; OR = 1.228, 95%CI = 1.075‐1.394), and Their‐Cognition was further associated with memory obj‐SCD (OR = 1.183, 95%CI = 1.020‐1.354). Descriptives in Table‐1, inferential results in Table‐2 and Figure‐1.

**Conclusion:**

Aβ‐pathology was linked to memory, but not global obj‐SCD, in participants not yet meeting MCI criteria. Subjective reports from informants showed greater effect sizes than reports from participants and were associated to memory and global obj‐SCD. The participant‐informant discrepancy observed for memory obj‐SCD and its association to AD‐related impairment suggested that participants with objective memory problems might not be fully aware of these subtle changes, highlighting the relevance of tracking dyadic reports in preclinical Alzheimer’s.